# Recurrent bilateral adrenal infarction with myelodysplastic/myeloproliferative neoplasm-unclassifiable (MDS/MPN-U): a case report

**DOI:** 10.1186/s12902-023-01384-5

**Published:** 2023-06-05

**Authors:** Yoshitomo Hoshino, Katsunori Manaka, Junichiro Sato, Yui Asatsuma, Hirofumi Horikoshi, Maki Takeuchi, Nobuaki Ito, Megumi Fujita, Megumi Yasunaga, Kensuke Matsuda, Akira Honda, Hiroaki Maki, Yosuke Masamoto, Mineo Kurokawa, Masaomi Nangaku, Noriko Makita

**Affiliations:** 1 Division of Nephrology and Endocrinology, The University of Tokyo Hospital, 7-3-1 Hongo, Bunkyo-Ku, Tokyo 113-8655 Japan; 2 Department of Hematology and Oncology, The University of Tokyo Hospital, Tokyo, Japan; 3 Department of Cell Therapy and Transplantation Medicine, The University of Tokyo Hospital, Tokyo, Japan

**Keywords:** Bilateral adrenal infarction, Recurrent adrenal infarction, Myelodysplastic/myeloproliferative neoplasm-unclassifiable (MDS/MPN-U), Primary adrenal insufficiency, Adrenocortical function, Case report

## Abstract

**Background:**

Bilateral adrenal infarction is rare and only a small number of cases have been reported so far. Adrenal infarction is usually caused by thrombophilia or a hypercoagulable state, such as antiphospholipid antibody syndrome, pregnancy, and coronavirus disease 2019. However, adrenal infarction with myelodysplastic/myeloproliferative neoplasm (MDS/MPN) has not been reported.

**Case presentation:**

An 81-year-old man with a sudden severe bilateral backache presented to our hospital. Contrast-enhanced computed tomography (CT) led to the diagnosis of bilateral adrenal infarction. Previously reported causes of adrenal infarction were all excluded and a diagnosis of MDS/MPN-unclassifiable (MDS/MPN-U) was reached, which was considered to be attributed to adrenal infarction. He developed a relapse of bilateral adrenal infarction, and aspirin administration was initiated. Partial primary adrenal insufficiency was suspected as the serum adrenocorticotropic hormone level was persistently high after the second bilateral adrenal infarction.

**Conclusion:**

This is the first case of bilateral adrenal infarction with MDS/MPN-U encountered. MDS/MPN has the clinical characteristics of MPN. It is reasonable to assume that MDS/MPN-U may have influenced bilateral adrenal infarction development, considering the absence of thrombosis history and a current comorbid hypercoagulable disease. This is also the first case of recurrent bilateral adrenal infarction. It is important to carefully investigate the underlying cause of adrenal infarction once adrenal infarction is diagnosed, as well as to assess adrenocortical function.

## Background

Bilateral adrenal infarction caused by venous adrenal gland thrombosis is rare [1]. The underlying diseases or conditions known to trigger adrenal infarction include antiphospholipid antibody syndrome (APS) [2–4], pregnancy [5–8], coronavirus disease 2019 (COVID-19) [9–11], and other thrombophilia or a hypercoagulable state. Adrenal infarction typically provokes backache and/or stomachache. It is diagnosed using characteristic imaging findings. It can be either unilateral or bilateral, and bilateral adrenal infarction can lead to primary adrenal insufficiency [2, 3]. Therefore, it is crucial to make a precise diagnosis of adrenal infarction.

Myelodysplastic/myeloproliferative neoplasm (MDS/MPN) has characteristics of both myelodysplastic syndrome (MDS), such as morphological dysplasia and ineffective hematopoiesis, and myeloproliferative neoplasm (MPN), such as the clonal proliferation of one or more hematopoietic cell lineages [12]. The World Health Organization (WHO) classification of tumors of hematopoietic and lymphoid tissues, 3rd edition, first introduced myelodysplastic/myeloproliferative disorders (MDS/MPD) and the nomenclature was changed to MDS/MPN in the WHO classification, 4th edition [13]. In the 2016 revision, MDS/MPN was newly classified into five types: chronic myelomonocytic leukemia; *BCR-ABL1*-negative atypical chronic myeloid leukemia; juvenile myelomonocytic leukemia; MDS/MPN with ring sideroblasts and thrombocytosis; and MDS/MPN-unclassifiable (MDS/MPN-U) [14].

We herein present a case of bilateral adrenal infarction with MDS/MPN-U, considered to be the underlying cause of adrenal infarction. Bilateral adrenal infarction relapsed over a relatively brief period of time, and partial primary adrenal insufficiency was highly suspected because high serum adrenocorticotropic hormone (ACTH) levels persisted. This is the first case of adrenal infarction with MDS/MPN-U as a possible prothrombotic complication, and the first case of recurrent bilateral adrenal infarction.

## Case presentation

**Fig. 1:**
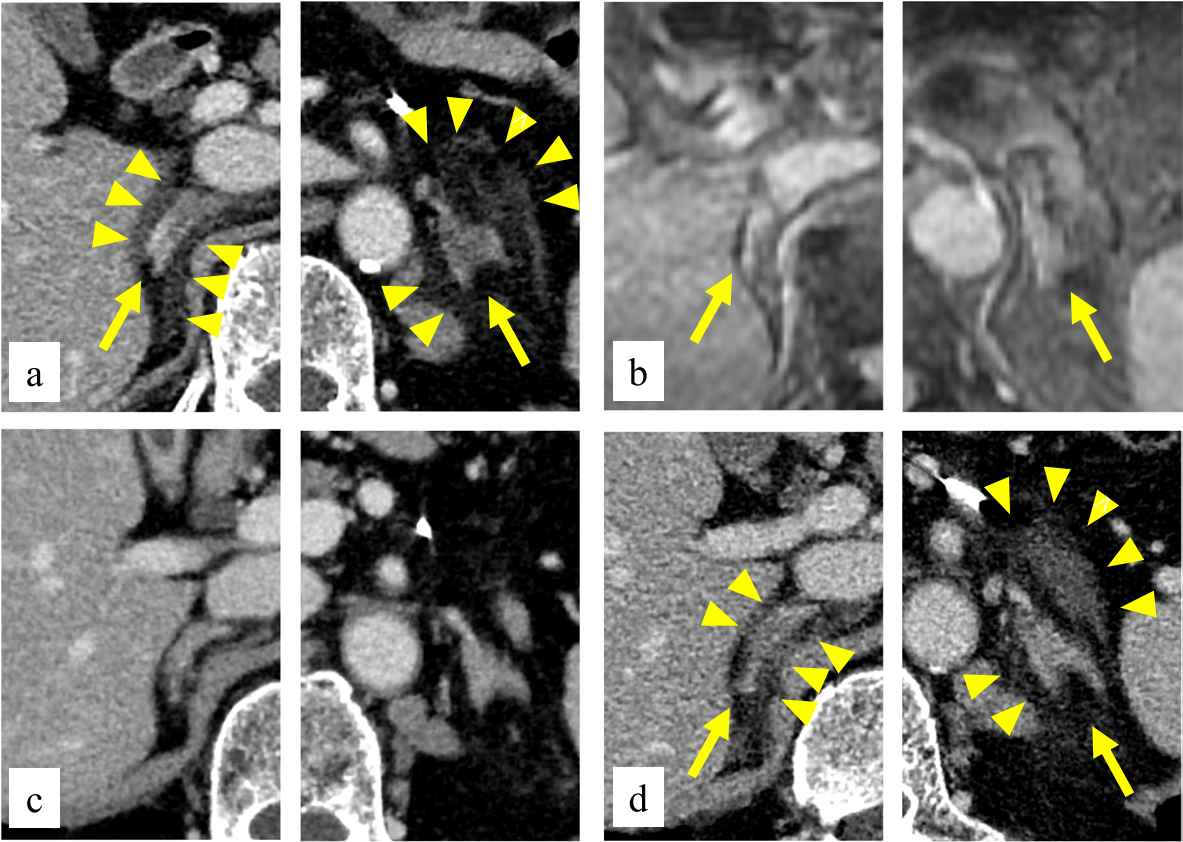
**a** Contrast-enhanced CT on day 1 showed bilateral adrenal hypertrophy (arrows), non-contrast-enhancing areas, and adjacent fatty inflammatory changes (arrowheads). Capsular signs were also observed. **b** No adrenal hemorrhage evidence was observed in T2 star-weighted MRI images on day 5 (arrows). **c** Contrast-enhanced CT on day 9 showed improvement in bilateral adrenal hypertrophy, non-contrast-enhancing areas, and adjacent fatty inflammatory changes. **d** Contrast-enhanced CT on day 62 demonstrated the same findings as on day 1, revealing bilateral adrenal infarction recurrence

An 81-year-old man presented to our hospital with a sudden severe bilateral backache. He had a medical history of bladder and prostate cancer at age 74 years and descending colon cancer at 80 years. These were completely cured by surgery. Postoperative surveillance with computed tomography (CT) imaging studies and colonoscopies showed no recurrence of these cancers. Bladder cancer surgery was performed along with a urostomy. Endocrine or hematologic disorders were negative on medical or family history. He had smoked 40 cigarettes per day for 45 years but quit at age 65 years. He consumed a small amount of alcohol once a week. Vital signs were normal. Physical findings were negative other than the severe back pain. He had no skin pigmentation, fatigue, postural hypotension, or recent weight loss. Contrast-enhanced CT was performed to examine the severe back pain, which showed bilateral adrenal hypertrophy, non-contrast-enhancing areas, and adjacent fatty inflammatory changes (Fig. 1a). This led to the diagnosis of bilateral adrenal infarction. The laboratory examination results (Table 1) revealed no evidence of primary adrenal insufficiency: the serum ACTH level was 34.64 pg/mL, serum cortisol 24.5 μg/dL, plasma renin activity (PRA) 0.2 ng/mL/h, and plasma aldosterone concentration (PAC) 3.34 ng/dL. Regarding PRA, he took Rikkunshito (oral Japanese herbal medicine), which contains Glycyrrhiza glabra before his admission. Its major ingredient is glycyrrhizin, and glycyrrhizin inactivates type 2 11β-hydroxysteroid dehydrogenase in the kidney, which can cause pseudoaldosteronism [15]. Although he did not show hypertension or hypokalemia, glycyrrhizin may have affected the suppression of renin. Catecholamine levels in the blood were high, reflecting his severe pain. He was admitted to our hospital for bilateral adrenal infarction and pain control. Analgesics were used as needed for back pain, but the back pain had completely disappeared by day 3 of admission. Abdominal magnetic resonance imaging (MRI) without contrast on day 5 demonstrated high signal intensity on diffusion-weighted imaging and no evidence of adrenal hemorrhage on T2 star-weighted imaging (Fig. 1b), consistent with adrenal infarction. Bilateral adrenal hypertrophy, non-contrast-enhancing areas, and adjacent fatty inflammatory changes had all improved (Fig. 1c) when contrast-enhanced CT was repeated on day 9.

**Table 1:** The patient’s laboratory data on admission

Parameter	Reference range	Value	Parameter	Reference range	Value
WBC (/μL)	3,300–8,600	1,800	ACTH (pg/mL)	8.7–61.5	34.64
Neutrophils (%)	40.0–75.0	55.0	Cortisol (μg/dL)	4.4–21.1	24.5
Lymphocytes (%)	16.5–49.5	25.0	DHEA-S (μg/dL)	5–253	152
Monocytes (%)	2.0–10.0	1.0	PRA (ng/mL/h)	0.2–3.9	0.2
Eosinophils (%)	0.0–8.5	19.0	PAC (ng/dL)	0–17	3.34
Basophils (%)	0.0–2.5	0.0	ADR (pg/mL)	0–100	1572
Blast (%)		0	NADR (pg/mL)	100–450	598
RBC (× 10^4^/μL)	435–555	263	DA (pg/mL)	0–20	25
MCV (fL)	83.6–98.2	103.8			
Hb (g/dL)	13.7–16.8	9.2	PT (%)	86.0–124.1	74.9
Plt (× 10^4^/μL)	15.8–34.8	46.8	APTT (%)	24–34	29.3
BUN (mg/dL)	8.0–20.0	11.8	Fbg (mg/dL)	168–355	303
Cre (mg/dL)	0.65–1.07	0.82	D-dimer (μg/mL)	0.0–1.0	2.8
Na (mEq/L)	138–145	136	PIC (μg/mL)	0.0–0.7	0.7
K (mEq/L)	3.6–4.8	3.9	TAT (ng/mL)	0.0–3.9	2.2
Cl (mEq/L)	101–108	100			
CRP (mg/dL)	0–0.3	0.33			

*WBC* white blood cell count, *RBC* red blood cell count, *MCV* mean corpuscular volume, *Hb* hemoglobin, *Plt* platelet, *BUN* blood urea nitrogen, *Cre* creatinine, *CRP* C-reactive protein, *ACTH* adrenocorticotropic hormone, *DHEA-S* dehydroepiandrosterone sulfate, *PRA* plasma renin activity, *PAC* plasma aldosterone concentration, *ADR* adrenaline, *NADR* noradrenaline, *DA* dopamine, *PT* prothrombin time, *APTT* activated partial thromboplastin time, *Fbg* fibrinogen, *PIC* alpha2-plasmin inhibitor–plasmin complex, *TAT* thrombin–antithrombin complex

Subsequent detailed examinations for thrombophilia or a hypercoagulable state were performed to search for the underlying cause of adrenal infarction, but there were no abnormal findings, as shown in Table 2. Although venous thrombosis, not arterial embolism, is the general cause of adrenal infarction, he had no previous history of atrial fibrillation, and he had neither fever nor heart murmur on his admission, which was not suggestive of endocarditis. However, peripheral blood tests revealed leukopenia and anemia, as well as the existence of giant platelets and blast cells. He was temporarily discharged from the hospital and further hematological examination was planned as his bilateral backache had disappeared and no further problems regarding adrenal glands occurred.

**Table 2:** Examinations for thrombophilia or a hypercoagulable state that can be the underlying cause of adrenal infarction

Clinical condition	Findings
Antiphospholipid antibody syndrome	Negative lupus anticoagulant
	Negative anti-cardiolipin IgG antibody
	Negative anti-cardiolipin and anti-β2-glycoprotein I antibody
Decrease in anticoagulant factors	Normal activities of proteins C and S
	Normal level of antithrombin III
COVID-19 infection	Negative PCR test for SARS-CoV-2
Heparin-induced thrombocytopenia	Negative heparin-induced thrombocytopenia antibody
Others	Normal activity of factor VIII
	No prolonged immobility
	No findings of malignancies

*COVID-19* coronavirus disease 2019, *PCR* polymerase chain reaction, *SARS-CoV-2* severe acute respiratory syndrome coronavirus 2

On day 62, however, he visited our hospital again with a chief complaint of stomachache and backache. Contrast-enhanced CT on reexamination showed bilateral adrenal hypertrophy, non-contrast-enhancing areas, and adjacent fatty inflammatory changes (Fig. 1d), which were the same as the previous CT on day 1. He was diagnosed with bilateral adrenal infarction recurrence and was readmitted. A laboratory examination on day 63 showed high platelet counts (619,000/μL) as well as persistent leukopenia and anemia (Table 3), and bone marrow examination was performed on day 65, with suspected hematological disease. Bone marrow findings revealed dysplasia in three major hematopoietic cell lineages, the presence of 4.2% blast cells, and megakaryocyte proliferation. No ring sideroblast was found. *BCR-ABL1*, *JAK2*, *CALR*, *MPL*, and *PDGFRA* genes, which can be detected in MPN genetic testing, were negative for mutations. Finally, he met the diagnostic criteria [16] and was diagnosed with MDS/MPN-U. A 24-h urinary free cortisol performed on day 99 was 38.5 μg/day, which was not significantly low, but high serum ACTH levels persisted even after the stomachache and backache improved (Table 3). There were no symptoms or findings suspicious of adrenal insufficiency, such as fatigue, hyponatremia, hyperkalemia, hypoglycemia, or hypotension. Partial primary adrenal insufficiency due to recurrent adrenal infarction was considered as the serum ACTH level was persistently high, although the rapid ACTH stimulation test was not performed because of concerns of the adrenal hemorrhage risk owing to adrenal blood flow increase [17]. He was directed to take oral hydrocortisone if he experienced illness or fever as a sick-day rule. Adrenal infarction treatment and intercurrent MDS/MPN-U were initiated with low-dose aspirin from day 68. Azacitidine for MDS/MPN-U was considered hereafter [18], although there was no established therapy for MDS/MPN-U.

**Table 3:** Clinical course and changes in hormone regarding adrenocortical function and complete blood count

	Reference range	Day 1	Day 8	Day 28	Day 62	Day 65	Day 77	Day 99	Day 139
Event		First bilateral AI			Second bilateral AI	BME			
ACTH (pg/mL)	8.7–61.5	34.64	58.18	82.22	32.53	N/A	78.72	100.80	131.99
Cortisol (μg/dL)	4.4–21.1	24.5	11.5	12.8	15.2	N/A	13.8	15.2	13.7
WBC (/μL)	3,300–8,600	1,800	2,600	1,800	2,500	7,000	3,200	2,400	N/A
Hb (g/dL)	13.7–16.8	9.2	7.9	8.7	8.1	8.6	8.5	7.7	N/A
Plt (× 10^4^/μL)	15.8–34.8	46.8	31.5	51.5	61.9	53.2	62.9	49.0	N/A

The values of complete blood count listed in day 62 are those of day 63. Administration of aspirin was initiated at day 68

*AI* adrenal infarction, *BME* bone marrow examination, *ACTH* adrenocorticotropic hormone, *WBC* white blood cell count, *Hb* hemoglobin, *Plt* platelet, *N/A* not available

## Discussion and conclusions

Bilateral adrenal infarction is a rare disease, and only a small number of cases have been reported in the context of APS [2, 3]. Waterhouse–Friderichsen syndrome is also known as a rare cause of bilateral adrenal hemorrhage [19, 20], in which bilateral adrenal vein thrombosis as the primary event may result in bilateral hemorrhagic adrenal gland infarction [2, 17]. However, the adrenal gland is known as an organ that is vulnerable to infarction and hemorrhage, primarily because of anatomic reasons. The adrenal gland has three arterial supplies: the superior adrenal artery, the middle adrenal artery, and the inferior adrenal artery. They form a plexus near the zona reticularis after branching into small arteries. They end abruptly [21] and venous drainage is provided by a single vein. The abrupt flow changes in the plexus creates hemodynamic turbulence, leading to platelet aggregation and adrenal infarction [4, 22], especially in thrombophilia or a hypercoagulable state. These anatomic characteristics are often described as a “vascular dam” [3, 21]. A previous study examined 32 cases of adrenal infarction, which reported that thrombosis occurred in the intra-adrenal muscular veins, emissary veins, venous sinuses, and/or capsular veins [1]. This study can support the above-mentioned theory that adrenal thrombosis occurs in the intra-adrenal veins.

Adrenal infarction often provokes a backache and/or a stomachache, but the clinical signs and symptoms are not specific. Therefore, it is important to carefully assess the imaging findings of the adrenal glands in such cases. Acute adrenal ischemia consists of adrenal hypertrophy, non-contrast-enhancing adrenal gland areas, and adjacent fatty inflammatory changes, which are classical imaging signs [6]. In addition, a peripheral subtle hyperdense line around a hypodense enlarged adrenal gland in contrast-enhanced CT is called a “capsular sign.” This was reported to have 83% sensitivity and 100% specificity for acute adrenal infarction [23]. The capsular sign probably reflects the residual perfusion within the adrenal capsular veins of the ischemic gland [6]. All these CT findings were observed in the present case. Acute adrenal infarction MRI findings demonstrate a high signal intensity on T2-weighted and diffusion-weighted imaging, as well as enlarged adrenal glands [9].

APS, pregnancy, COVID-19, and other hypercoagulable states, which have been reported as causes of adrenal infarction, were all excluded in this case. Our patient had a medical history of malignancies, and although malignancies can cause hypercoagulable states [24], follow-up imaging studies and colonoscopy showed no signs of recurrence.

There is neither established evidence that MDS/MPN is prone to thrombosis nor reports of adrenal infarction with MDS/MPN. A previous report suggested that MDS/MPN may be prone to venous thromboembolism [25]. However, MDS/MPN has clinical MPN characteristics by definition. MPN includes essential thrombocythemia, polycythemia vera, and primary myelofibrosis, which have been reported to increase the risk of thrombotic events [26]. There are a few previous reports of adrenal infarction in the MPN setting [27, 28]. It is reasonable to assume that MDS/MPN-U may have influenced the development of bilateral adrenal infarction considering the absence of thrombosis history and a current comorbid hypercoagulable disease. MDS/MPN-U has a clinical MPN feature, and age > 60 years is a significant risk factor for thrombosis in MPN [29, 30]. Older age in this case may have greatly increased the risk of MDS/MPN-U thrombosis, although the platelet count was only slightly elevated. This is the first case of adrenal infarction in the setting of MDS/MPN-U.

This is also the first case with a recurrence of bilateral adrenal infarction over a relatively brief period of time. There are some case reports in which anticoagulants were administered in accordance with the underlying diseases or conditions, although there is no evidence-based treatment for adrenal infarction itself. Heparin, for example, was administered in cases of adrenal infarction with APS [2, 3] or pregnancy [5, 7, 8], and warfarin was administered in a case of COVID-19 [11]. Attention should be paid to the consequent adrenal hemorrhage [3, 22] once anticoagulation therapy is initiated. On the other hand, aspirin was used to prevent thrombosis in cases of MPN [28]. Our patient was closely followed without treatment for adrenal infarction. Initially, it was because the underlying disease of adrenal infarction was not elucidated in the first adrenal infarction. However, as a result of repeated adrenal infarction leading to the MDS/MPN-U diagnosis, aspirin administration was initiated to prevent further adrenal infarction and other thromboses.

Bilateral adrenal infarction can cause primary adrenal insufficiency when more than 90% of the cortex is destroyed [2]. Adrenal insufficiency caused by bilateral adrenal infarction/hemorrhage is generally irreversible, although there is some improvement in adrenal function in some cases [2, 31]. Corticosteroid replacement therapy is indispensable in cases of bilateral adrenal infarction with primary adrenal insufficiency. Hence, it is worthy of consideration to assess adrenocortical function by performing the rapid ACTH stimulation test [3] after the bilateral adrenal infarction diagnosis, but there would be a risk of adrenal hemorrhage associated with increased adrenal blood flow due to ACTH stimulation [17]. Careful follow-up is important in patients with adrenal infarction to assess adrenocortical function as adrenal hemorrhage occurs at the time of reperfusion of necrotic vessels at the site of adrenal infarction [22], and it could result in evoking newly developed adrenal insufficiency.

In conclusion, we report a rare case of bilateral adrenal infarction with MDS/MPN-U relapse over a relatively brief period of time. Examination for the cause of adrenal infarction did not reveal any previously reported findings of thrombophilia or a hypercoagulable state, so MDS/MPN-U was attributed to adrenal infarction in this case. It is important to investigate the underlying cause of adrenal infarction carefully once adrenal infarction is diagnosed, as well as to assess adrenocortical function.

## Data Availability

All relevant data are included in the manuscript.

## Abbreviations

MDS/MPN: Myelodysplastic/myeloproliferative neoplasm
CT: Computed tomography
MDS/MPN-U: MDS/MPN-unclassifiable
ACTH: Adrenocorticotropic hormone
APS: Antiphospholipid antibody syndrome
COVID-19: Coronavirus disease 2019
PRA: Plasma renin activity
PAC: Plasma aldosterone concentration
MRI: Magnetic resonance imaging

## References

[1] Fox B (1976). Venous infarction of the adrenal glands. J Pathol.

[2] Ramon I, Mathian A, Bachelot A, Hervier B, Haroche J, Boutin-Le Thi Huong D, et al. Primary adrenal insufficiency due to bilateral adrenal hemorrhage-adrenal infarction in the antiphospholipid syndrome: long-term outcome of 16 patients. J Clin Endocrinol Metab. 2013;98(8):3179–89. 10.1210/jc.2012-4300 23783099

[3] You JY, Fleischer N, Abraham SB (2019). Evolving adrenal dysfunction after bilateral adrenal infarction: a case report. AACE Clin Case Rep.

[4] Riddell AM, Khalili K (2004). Sequential Adrenal Infarction Without MRI-Detectable Hemorrhage in Primary Antiphospholipid-Antibody Syndrome. Am J Roentgenol.

[5] Chagué P, Marchi A, Fechner A, Hindawi G, Tranchart H, Carrara J (2021). Non-Hemorrhagic Adrenal Infarction during Pregnancy: The Diagnostic Imaging Keys. Tomography.

[6] Sidibe S, Perazzini C, Cassagnes L, Boyer L, Magnin B (2021). The role of computed tomography in adrenal gland infarction diagnosis during pregnancy: Two case reports. J Med Vasc.

[7] Sormunen-Harju H, Sarvas K, Matikainen N, Sarvilinna N, Laitinen EK (2016). Adrenal infarction in a healthy pregnant woman. Obstet Med.

[8] Green PA, Ngai IM, Lee TT, Garry DJ. Unilateral adrenal infarction in pregnancy. BMJ Case Rep. 2013;2013:bcr2013009997. 10.1136/bcr-2013-009997 PMC3762357 23975911

[9] Machado IFR, Menezes IQ, Figueiredo SR, Coelho FMA, Terrabuio DRB, Ramos DV, et al. Primary adrenal insufficiency due to bilateral adrenal infarction in COVID-19: a case report. J Clin Endocrinol Metab. 2022;107(1):e394–e400. 10.1210/clinem/dgab557 34324679

[10] Leyendecker P, Ritter S, Riou M, Wackenthaler A, Meziani F, Roy C (2021). Acute adrenal infarction as an incidental CT finding and a potential prognosis factor in severe SARS-CoV-2 infection: a retrospective cohort analysis on 219 patients. Eur Radiol.

[11] Kumar R, Guruparan T, Siddiqi S, Sheth R, Jacyna M, Naghibi M (2020). A case of adrenal infarction in a patient with COVID 19 infection. BJR Case Rep.

[12] Cazzola M, Malcovati L, Invernizzi R (2011). Myelodysplastic/myeloproliferative neoplasms. Hematol Am Soc Hematol Educ Program.

[13] Vardiman JW, Thiele J, Arber DA, Brunning RD, Borowitz MJ, Porwit A (2009). The 2008 revision of the World Health Organization (WHO) classification of myeloid neoplasms and acute leukemia: rationale and important changes. Blood.

[14] Arber DA, Orazi A, Hasserjian R, Thiele J, Borowitz MJ, Le Beau MM (2016). The 2016 revision to the World Health Organization classification of myeloid neoplasms and acute leukemia. Blood.

[15] Yoshino T, Shimada S, Homma M, Makino T, Mimura M, Watanabe K (2021). Clinical Risk Factors of Licorice-Induced Pseudoaldosteronism Based on Glycyrrhizin-Metabolite Concentrations: A Narrative Review. Front Nutr.

[16] Shallis RM, Zeidan AM (2020). Myelodysplastic/myeloproliferative neoplasm, unclassifiable (MDS/MPN-U): More than just a "catch-all" term?. Best Pract Res Clin Haematol.

[17] Godfrey RL, Clark J, Field B. Bilateral adrenal haemorrhagic infarction in a patient with antiphospholipid syndrome. BMJ Case Rep. 2014;2014:bcr2014207050. 10.1136/bcr-2014-207050 PMC4244402 25410037

[18] Talati C, Padron E (2016). An Exercise in Extrapolation: Clinical Management of Atypical CML, MDS/MPN-Unclassifiable, and MDS/MPN-RS-T. Curr Hematol Malig Rep.

[19] Tourrel F, Gouin P, Dureuil B, Veber B (2007). Waterhouse-Friderichsen syndrome associated to a Morganella morganii and Enterococcus faecium peritonitis. Ann Fr Anesth Reanim.

[20] Pereira FDA, Hickson ML, Wilson PAJ (2019). Case 268: Bilateral Adrenal Hemorrhage in the Context of Sepsis. Radiology.

[21] Fowler AM, Burda JF, Kim SK (2013). Adrenal artery embolization: anatomy, indications, and technical considerations. AJR Am J Roentgenol.

[22] Galatola R, Gambardella M, Mollica C, Calogero A, Magliulo M, Romeo V (2020). Precocious ischemia preceding bilateral adrenal hemorrhage: A case report. Radiol Case Rep.

[23] Moschetta M, Telegrafo M, Pignatelli A, Stabile Ianora AA, Angelelli G (2015). Value of the CT "capsular sign" as a potential indicator of acute adrenal ischemia. Emerg Radiol.

[24] Caine GJ, Stonelake PS, Lip GY, Kehoe ST (2002). The hypercoagulable state of malignancy: pathogenesis and current debate. Neoplasia.

[25] Péan de Ponfilly-Sotier M, Jachiet V, Benhamou Y, Lahuna C, De Renzis B, Kottler D, et al. Venous thromboembolism during systemic inflammatory and autoimmune diseases associated with myelodysplastic syndromes, chronic myelomonocytic leukaemia and myelodysplastic/myeloproliferative neoplasms: a French multicentre retrospective case-control study. Clin Exp Rheumatol. 2022;40(7):1336–42. 10.55563/clinexprheumatol/nbn38d 35579092

[26] Hultcrantz M, Björkholm M, Dickman PW, Landgren O, Derolf ÅR, Kristinsson SY (2018). Risk for Arterial and Venous Thrombosis in Patients With Myeloproliferative Neoplasms: A Population-Based Cohort Study. Ann Intern Med.

[27] Michiels JJ, Berneman Z, Schroyens W, Krestin GP (2002). Bilateral adrenal swelling as a cause of chest, back, and upper abdominal pain in essential thrombocythemia and polycythemia vera is due to microvascular ischemic thrombosis rather than to hemorrhage. Ann Hematol.

[28] Burnet G, Lambert M, Annet L, Lefebvre C (2015). Unilateral adrenal hemorrhagic infarction in essential thrombocythemia. Acta Clin Belg.

[29] Barbui T, Barosi G, Birgegard G, Cervantes F, Finazzi G, Griesshammer M (2011). Philadelphia-negative classical myeloproliferative neoplasms: critical concepts and management recommendations from European LeukemiaNet. J Clin Oncol.

[30] Barbui T, Finazzi G, Carobbio A, Thiele J, Passamonti F, Rumi E, et al. Development and validation of an International Prognostic Score of thrombosis in World Health Organization-essential thrombocythemia (IPSET-thrombosis). Blood. 2012;120(26):5128–33; quiz 252. 10.1182/blood-2012-07-444067 23033268

[31] Jahangir-Hekmat M, Taylor HC, Levin H, Wilbur M, Llerena LA (2004). Adrenal insufficiency attributable to adrenal hemorrhage: long-term follow-up with reference to glucocorticoid and mineralocorticoid function and replacement. Endocr Pract.

